# Clinical Outcome of Transfemoral Direct Socket Interface (Part 2)

**DOI:** 10.33137/cpoj.v4i1.36065

**Published:** 2021-06-08

**Authors:** J. Walker, W.R. Marable, C. Smith, B.Þ Sigurjónsson, I.F. Atlason, G.A. Johannesson

**Affiliations:** 1 Virginia Prosthetic & Orthotics, Roanoke, Virgina, USA.; 2 össur HF, Foothill Ranch, California, USA.; 3 össur HF, Reykjavik, Iceland.; 4 Quick Lookup, Reykjavik, Iceland.; 5 TeamOlmed, Stockholm, Sweden.

**Keywords:** Transfemoral Amputation, Health, Amputee, Prosthesis, Socket, Interface, Comfort, Outcome Measure, Satisfaction, Direct Casting

## Abstract

**BACKGROUND:**

Amputation at the transfemoral (TF) level reduces the rate of successful prosthetic fitting, functional outcome, and quality of life (QoL) compared with transtibial amputation. The TF socket interface is considered the most critical part of the prosthesis, but socket discomfort is still the most common user complaint. Direct Socket for transfemoral prosthesis users is a novel interface fabrication process where the socket is shaped and laminated directly on the residual limb and delivered in a single visit.

**OBJECTIVES:**

The aim of this study was to investigate if prosthetic users’ quality of life (QoL), comfort, and mobility with a Direct Socket TF interface were comparable to their experience with their previous prostheses.

**METHODOLOGY:**

The pre/post design prospective cohort study included 47 subjects. From this cohort, 36 subjects completed the 6-months follow-up (mean age 58 years, 27 males). Outcomes at baseline included EQ-5D-5L^®^, PLUS-M™, CLASS, ABC, AMPPRO, and TUG. At 6-weeks and 6-months, subjects repeated all measures. Seven Certified Prosthetist (CP) investigators performed observations and data collection at six different sites (from July 2018 to April 2020).

**FINDINGS:**

Results showed significant improvement in all outcome measures for the 36 subjects that completed both 6-weeks and 6-months follow-ups. CLASS sub-scales showed significantly improved stability, suspension, comfort, and socket appearance. Improvement in K-Level and less use of assistive devices were observed with the AMPPRO instrument, indicating improved user mobility and performance. QoL was also increased, as measured in Quality-Adjusted-Life-Years (QALY) from the EQ-5D-5L.

**CONCLUSIONS:**

Evidence from the findings demonstrate that the Direct Socket TF system and procedure can be a good alternative to the traditional method of prosthetic interface delivery.

## INTRODUCTION

The primary goal for people that have undergone lower limb amputation is to return to the main activities of daily living, including recreational and professional activities.^1^ Transfemoral (TF) amputation and the subsequent loss of knee function is known to negatively impact prosthetic fitting, functional outcome, and quality of life (QoL), as compared to transtibial amputation.^2–7^ Persons with TF amputation face significantly more challenges when receiving a prosthesis for the first time. With more days spent in rehabilitation, the functional outcome and QoL are still lower at the time of discharge compared to persons with transtibial (TT) amputation.^7–11^ Additionally, the Certified Prosthetist (CP) faces a more significant challenge in fitting TF patients versus TT patients.^3,12^ TF amputations may account for approximately 40% of all lower-limb amputations in the US alone and result in a twofold higher mortality rate than after TT amputations^13^ and a lower rate of prosthetic fitting.^5^ Also, unfortunately, most CPs have less experience fitting TF amputees; thus, outcomes and proficiency are diminished.^14^ Moreover, TF interfaces are researched less frequently than TT interfaces. Therefore, CPs have less published evidence to guide their practice.^12^ A meager body of published evidence surrounds TF interfaces, and most lack methodological quality. Investigations assessing the advantages and disadvantages of available socket designs are few and lack randomized controls.^12^ The heterogeneity of intervention, study population, and outcome measures make meta-analysis impossible.^3,12,14,15^

The TF socket interface, considered the most important part of the prosthesis, allows the user to control the prosthesis and provides pelvic stabilization during loading. The ischial ramus containment (IRC) socket claims to feature a “bony lock” believed to be most effective during full stance phase. The “bony lock” is defined as a 3-point support between the femoral shaft, the ischium, and a high medial and lateral socket trimline.^16^ While we can observe anecdotally that many patients succeed with such socket designs, only theoretical models and limited evidence exist to support 3-point support efficacy.^17^ Despite limited evidence, the "3-point support" sockets enjoy wide popularity, and many western countries consider them the standard of care, especially for active users. However, based on different functional philosophies, alternative socket designs are available. Performance can be retained, and comfort increased while abandoning or altering the 3-point support^17–19^

Furthermore, traditional fabrication and delivery of TF interfaces often involve multiple client visits to the prosthetic clinic for casting or scanning, diagnostic interface (or “check socket”) fittings and modifications, definitive laminated interface fabrication, and final fitting and delivery.^2,11,20^ The user then returns as needed for adjustments and modifications mostly related to comfort and functional issues. It is understood that the sooner a patient can begin gait training with their definitive socket, the better.^21,22^

The Direct Socket TF (DS-TF) enables a prosthetist to fabricate a custom-made interface directly on the residual limb in a single visit, similar to Direct Socket TT.^23^ The proximal portion of a DS-TF interface design differs from the proximal portion of sockets, typically included in the above described IRC sockets designs,^16^ as the proximal part of the DS-TF incorporates a size-specific silicone brim instead. The support provided by the DS-TF brim activates and stimulates important hip muscle function^24^ to enable loading and axial/transverse stability during normal walking.^23^ The unique DS-TF interface design has led to greater user satisfaction regarding interface function, comfort, and overall improvement in the fabrication and delivery experience compared to traditional methods.^25–27^ A deeper analysis of the outcome requires more specific tools.^12^

Dissonance between the user and the prosthetic interface design can negatively impact comfort level and strongly correlates with lower functional outcomes.^14^ It often leads to increased residual pain, phantom pain,^28^ restricted movement,^29^ dermatological problems, or a combination of those,^30^ most often related to the proximal trimline and lack of femur stability and positioning. Historically the use of Self-report instruments has not been the standard in daily practice, but the prevalence of their application has been increasing.^31,32^ CPs are improving their implementation of such instruments to help screen amputees for prosthetic fitting candidacy and monitor mobility and comfort outcomes.^33^ Responses facilitate communication between the prosthetic user and clinician, inform training decisions, and evaluate care efficacy.^34,35^ CPs have access to self-report instruments, specifically applicable to TF prosthetic users that measure health outcomes, socket comfort, and mobility. Combining self-report and performance-based evaluation measures reveals a comprehensive picture of overall function. This type of detailed data is increasingly used to inform healthcare policies and payments.^36^

This paper investigates if DS-TF direct lamination,^25^ in contrast to traditional plaster casting and 3D scanning techniques, can result in similar outcomes for the end-user. In this way, using plaster or foam model intermediates, that are only an approximation of the limb shape, are eliminated. We compared the new DS-TF interface versus the existing traditional socket using two modules from the Orthotics and Prosthetics ‘User’s Survey (OPUS) in the first article on this study.^27^ This paper, aiming to build on the previous publication, quantifies different subjective and objective outcomes using instruments applicable to this specific population of prosthetic users.

## METHODOLOGY

Between July 2018 and October 2019, seven CPs in six prosthetic clinics across the United States implemented a new prosthetic interface for transfemoral prosthesis users. In total, 47 subjects were enrolled into this study. The previous article was derived from the same cohort^27^ and includes detailed descriptions of principal investigator selection criteria, practitioner/clinician training, implementation process, and interface design. The inclusion criteria (rationale) were as follows:

- 50Kg<bodyweight<166Kg (the ISO validated weight limit of the DS-TF)
- Cognitive ability to understand all instructions and questionnaires in the study
- Patients who have undergone a TF amputation>1 year post-amputation (this was to avoid postoperative problems and/or adjustments related to the initial prosthetic fitting of a new amputee)
- Willing and able to participate in the study and follow the protocol
- Circular dimension of 40-65 cm at the crotch (limited to available silicone brim sizes)
- Residual limb length at least 20 cm from ischium to the distal end (fabrication limitation of the DS-TF)
- Currently using a prosthetic liner (this was to avoid potential confounding influence from transitioning an amputee from a skin fitting interface (i.e., without a liner) to an interface with a liner)

**Figure 1:**
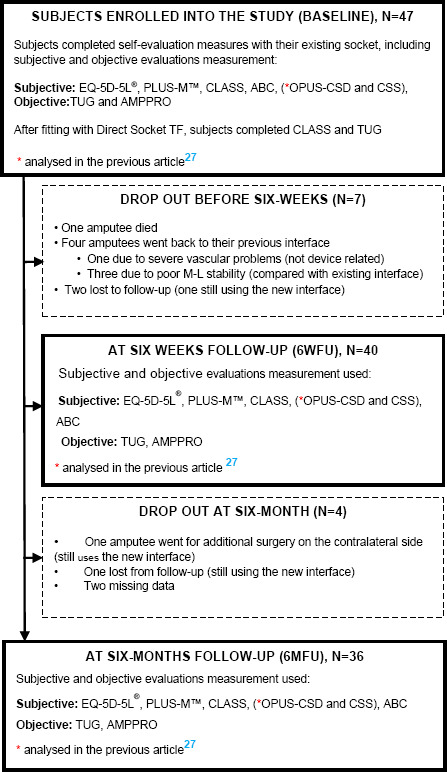
Flow chart of the trial.

Ethical approval was obtained from Advarra^®^ IRB (CR00128417), and the investigation was registered at Clinicaltrials.gov NCT04312724. Signed informed consent was obtained from all participants. All study subjects completed the baseline measurements related to their current prosthesis (**Figure 1**). Each subject was consequently fitted with DS-TF by one CP with one Technician’s assistance. Two subjects received a new knee and foot with the new interface.

### Manufacture and features of the DS-TF

During the fabrication process, a specific casting liner is rolled onto the residual limb, followed by application of a protective silicone sheath. Next, the CP places a size-specific silicone brim at the proximal part of the limb. A fiberglass or basalt fabric with a pre-attached 4-hole distal adapter is then rolled on the length of the limb. An additional protective sheath is applied on the outside of the fabric, and a two-part resin is injected through the distal adapter. The resin saturates the fiber, and then it hardens and cures. After 10-15 minutes it has cured enough to be removed. Finally, the socket is prepared to be connected to their knee and foot.^25,26^

The brim is made of flexible silicone and laminated to the socket during this process, making the socket flexible proximally, while most of the socket is rigid. The flexible silicone brim encompasses and compresses the proximal thigh muscles when contracted, thereby stabilizing the hip at initial contact, loading response, mid-stance, and terminal-stance while creating axial and transverse stabilization.^27^ During swing phase, the brim only follows the hip movement.

### Outcome measures and data Collection

Each prosthesis user completed two parts of the Orthotics and Prosthetics User’s Survey (Client Satisfaction with Device (OPUS CSD) and Client Satisfaction with Services (OPUS CSS)) for evaluating the new interface and the service model. Additional data was collected using multiple standardized outcome measures, including:

- *Four subjective self-evaluation measures:* health status; perceived mobility level; satisfaction regarding socket stability, suspension, comfort, and appearance; and confidence regarding balance and perceived risk of falls.
- *Two objective performance measures*.

Most outcome measures (PLUS-M(tm),^37^ CLASS,^38^ ABC,^39^ TUG,^40^ and AMPPRO^41^) were collected using the Prosthetic Rehabilitation Outcomes Application (PROApp) iPad application. Two measures were collected using paper forms (EQ-5D-5L^®^ and OPUS). The PROApp systematically helps the clinician gather and securely store subjective and functional outcomes data.

The PROApp also helps with clinical care decisions, communication with referral sources, physical therapy, and validating prosthetic care to payor sources.^42^ **Table 1** contains full description of the outcome measures used. Outcome measures were collected on each subject at three time periods: baseline, 6-weeks post fitting, and 6-months post fitting. Subject completed CLASS, Plus-M, ABC, AmpPro, OPUS, EQ-5D, and TUG using their existing socket. On the day of fitting, subjects completed TUG (as an objective measurement) and CLASS (as a subjective measurement) with their new DS-TF interface (**Figure 1**). Subjects returned to repeat CLASS, Plus-M, ABC, AmpPro, OPUS, EQ-5D, and TUG six weeks after fitting and then again six months after fitting. The goal was to collect 940 datasets: meaning every subject (N=47) completed 20 outcome measurements in total over the six months.

**Table 1:** Full description of the outcome measures used in this study.

•	**EQ-5D-5L^®^**: A valid and reliable questionnaire to describe and value health. The descriptive system comprises five dimensions: mobility, self-care, usual activities, pain/discomfort, and anxiety/depression. Each dimension has 5 levels: o *No problems, Slight problems, Moderate problems, Severe problems and Extreme problems.* The patient is asked to indicate his/her health state by ticking the box next to the most appropriate statement in each of the five dimensions. This decision results in a 1-digit number that expresses the level selected for that dimension. The digits for the five dimensions can be combined into a 5-digit number that describes the ‘patient’s health state. This health state is then an indicator of the utility of the patient. One of the values that can be derived from EQ-5D-5L^®^ is quality-adjusted life-year (QALY).^43^ The mean (standard deviation) utility value for the US population in 2020 was estimated to be 0.85 (SD=0.21).^43^ QALYs can be used to evaluate the efficacy of one healthcare intervention versus another. QALY can be used to guide patients and providers to prosthetic solutions that maximize QoL increases while also implementing efficient fitting processes and using healthcare funds responsibly.^44^
•	**PLUS-M(tm):** The Prosthetic Limb Users Survey of Mobility (PLUS-M(tm)) measures perceived mobility and was developed for lower limb prosthesis users. The survey includes 12 questions that assess mobility with a prosthetic leg and are answered on a five-point scale ranging from “unable to do” the activity, to able to do the activity "without any difficulty". A higher PLUS-M(tm) T-score corresponds to greater mobility. PLUS-M(tm) T-scores are referenced to the PLUS-M(tm) development sample (n=1091 lower limb prosthesis users). A T-score has a mean of 50 and a standard deviation of 10. A PLUS-M(tm) T-score of 50 represents the mean mobility reported by the development sample. A respondent that receives a T-score of 60 has reported a level of mobility approximately 1 standard deviation above the mean. Conversely, a respondent that receives a T-score of 40 has reported their mobility to be about one standard deviation below the mean.^45^
•	**CLASS:** The Comprehensive Lower-limb Amputee Socket Survey is a newly developed outcome measurement tool. It reports measures regarding the function of the prosthetic interface (e.g., the socket) that is composed of 15 items. The first five context items are scored using a 4-point Likert-type scale with response options and the corresponding point value of: strongly disagree (1), disagree (2), agree (3), and strongly agree (4). The last item of every determinant addressed the overall satisfaction and was scored using a numerical rating scale with values ranging between 0 and 5 points. The maximum possible score for each of the four determinants was 25 points. The CLASS score for each subscale is then represented on a 0%–100% scale (with 100% indicating excellent satisfaction). The average overall socket satisfaction including all levels (ankle, transtibial, knee and transfemoral) of the CLASS, administered measured on 124 LLA, was found to be for Stability 70% (SD=18), Suspension 71% (SD=19), Comfort 69% (SD=20) and Appearance 58% (SD=22).^15^
•	**ABC**: Activities Based Confidence indicator quantifies ‘individuals’ confidence in their ability to perform 16 activities of daily living by rating confidence from 0% (no confidence) to 100% (complete confidence) for each activity. The scores for each of the activities are averaged to obtain a total score.^46^ In a study by Miller et.al., the mean score was 63.8. For subjects amputated due to vascular reasons, it was 54.1 and 74.7 for subjects with amputation due to non-vascular reasons.^39^
•	*OPUS: The OPUS (Orthotics and Prosthetics ‘User’s Survey) is a set of self-reported outcome measures to be used within O&P clinics for the assessment of functional status, quality of life, and client satisfaction and was used in the previous article.^27^ The OPUS instrument consists of five independent modules, two of which were used in this study: Client Satisfaction with Device (CSD) and Client Satisfaction with Services (CSS). The CSD and CSS include a total of 21 questions, scored on a 1-6, discrete scale: Strongly Agree, Agree, Neither agree nor disagree, Disagree, Strongly disagree and Don’t Know/Not Applicable.^47^ Different authors have used scoring for OPUS in a different way, so there is no minimal or maximal score reported. The mean score reported by Jarl et.al. for CSD was 36.5 and for CSS was 55.7 on a US sample of 126 prosthetic users (all levels).^48^
**Performance-based (objective) outcome measurements were:**	
•	**TUG**: The Timed Up and Go test is a test where the prosthetic user performs the test by standing up from a chair, walking ten feet, turning and returning to the chair, and then sit on the chair. This measure tests a number of tasks that are essential for mobility, such as standing from a seated position, walking, turning, and sitting down on the chair, and can be used with or without walking aid. A lower limb prosthetic user who takes ≥19 seconds to complete the TUG indicates an increased risk of fall^49^
•	**AMPPRO**: This clinical tool is designed for assessing an amputee ‘subject’s mobility and for assessing existing or potential functional ambulation of the lower-limb amputee. It consists of 21 tasks, classified into four categories: sitting balance, simple mobility, standing balance, and gait and functional activities. The total score ranges from 0 to 47 points. Higher scores indicate better mobility.^41^ The normative data for lower limb amputees has been established according to the K levels classification with the five categories ranging from K level 0 (least mobile/not using prosthesis) to K level 4 (most mobile) intended to indicate a ‘person’s rehabilitation potential,^35^ using the AMPPRO Score. The normative values of AMPPRO for each of the K Levels classification have been set to: • **K1** = AMPPRO 15-26, **K2** = AMPPRO 27-36, **K3** = AMPPRO 37-42,**K4** = AMPPRO 43-47

* Included in previous article^27^

### Sample size and statistical methods

We conducted a pretrial power analysis for the estimated required sample size using GPower version 3.1.9.6^50^ and estimated effect size based on published articles^7^,^51^,^52^ for the primary endpoint assuming a normally distributed amputee population. Therefore, we expected that 38 subjects were required to complete the protocol with a power of 0,95 and α at 0,05. We estimated the drop-out rate to be 20%, and therefore, 47 subjects were recruited.

We used R version 4.03 (R-Studio Version 1.2.5033) and lme4.^53^ We performed linear mixed-effects analyses of the relationship between outcomes and clinical need (defined as users who needed a replacement prosthetic interface, according to new referral, due to wear and tear or volume changes) and those with no clinical need of replacement. As fixed effects, we entered age, gender, and evaluation point (tested for interaction with clinical need) into the model. As random effects, we included intercepts for subjects and investigators and by-subject and by-item random slopes for the effect of clinical need. P-values were obtained by likelihood ratio tests of the full model with the effect in question against the model without the effect.

## RESULTS

**Figure 2:**
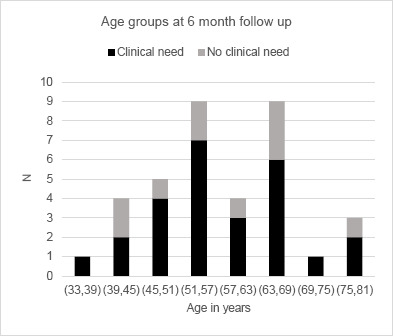
Age groups at 6MFU divided into “clinical need” (N=26) and “no clinical need” (N=10).

The subjects, with a mean age of 59 years (36-79 years) when entering the study, 33 men and 14 women prosthesis users, represented a wide range of activity levels (**Figure 2**).

Of the initial cohort, 36 participants completed the entire 6-month follow-up (6MFU; 27 men and 9 women: mean age 58.2 years (38-81 years). Nine subjects dropped out of the study: 7 at or before 6-week follow-up (6WFU; including one deceased) and 2 before 6MFU (**Figure 1**). One subject with advanced vascular disease and a small limb withdrew after one week. Three subjects withdrew, preferring their previous socket. Two subjects in the “clinical need” sub-group did not complete all measurements at the three time periods and therefore were not included in further data analysis at 6MFU. One subject became non-ambulatory for a portion of the study for reasons unrelated to the prosthesis. Though he was considered a drop-out, he continued to use the new interface after the study ended. Three subjects didn’t come in for follow-up measurements. At least two of them continued to use the new DS-TF interface. All subjects used a liner with their interface. A description of their prosthesis can be found in the previous study.^27^

Subjects represent the full spectrum of K-Levels 1 through 4:

- K-level 1, n=4
- K-level 2, n=11
- K-level 3, n=21
- K-level 4, n=11

Upon inclusion, participants were classified into two sub-groups: subjects with a “clinical need” of socket replacement and subjects with “no clinical need”, as determined by their CP. The mean age of the “clinical need” sub-group was 59.0 year (SD=11.8), and the mean age of the “no clinical need” sub-group was 58.8 year (SD=12.3). Mean ages in the two sub-groups remained comparable throughout the investigation period providing a power of 95.3% and 94.0% for the follow-ups, respectively (**Figure 2**).

After 6MFU, we had collected 694 datasets, out of a maximum potential of 720 datasets, from the 36 subjects that completed the follow-up. This gave us a 3.6% rate of missing data (**Figure 1**). Missed appointments and software error accounted for the following instances of missing data:

- At Baseline/delivery: 2 CLASS, 1 AMPPRO, and 2 TUG data sets
- At 6WFU: 2 PLUS-M(tm), 2 CLASS, 2 ABCs, 4 TUG and 3 AMPPRO
- At 6MFU: 2 EQ-5D-5L^®^, 1 PLUS-M(tm), 1 CLASS, 2 ABC, 1 TUG, and 2 AMPPRO

Outcome measure results are presented in three sections below. The first section presents data including all participants that completed the 6MFU. The following sections present findings from the two sub-groups within the cohort – “clinical need” and “no clinical need” sub-groups.

## Satisfaction and functional assessment of all participants (N=36) (Table 2A):

The EQ-5D-5L^®^ mean utility score was 0.75 using the existing prosthesis at baseline (SD=0.18). Life quality increased significantly to 0.82 (SD=0.15) at 6WFU and to 0.84 (SD=0.12) at 6MFU.

The PLUS-M(tm) score at baseline on the existing prosthesis was 46 (SD=24). Mean mobility scores rose significantly to 54 (SD=21) at 6WFU and to 61(SD=16) at 6MFU (e.g., >1 standard deviation above the mean after 6MFU).

**Figure 3:**
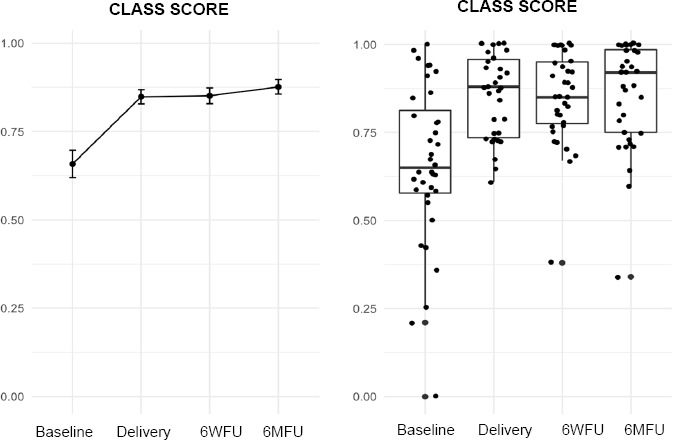
CLASS score at baseline (with existing socket) and at delivery of the new socket, including follow-up at 6 weeks and 6 months.

The CLASS mean overall score improved significantly from 74% at baseline with the existing interface to 86% on the day of fitting with DS-TF. This improvement was maintained during the follow-up period (**Figure 3**).

The cohort’s ABC mean score at baseline was 63% (SD=25) on the existing prosthesis. The scores rose significantly to 75% at 6WFU and further to 78% at 6MFU.

The TUG mean time at baseline on the existing prosthesis was 14.7sec. (SD=7.4) compared to 14.0 sec. (SD=6.5) with DS-TF. TUG times improved significantly to 13.0 sec. (SD=6.5) at 6WFU and 12.8 sec. (SD=6.5) at 6MFU.

**Figure 4:**
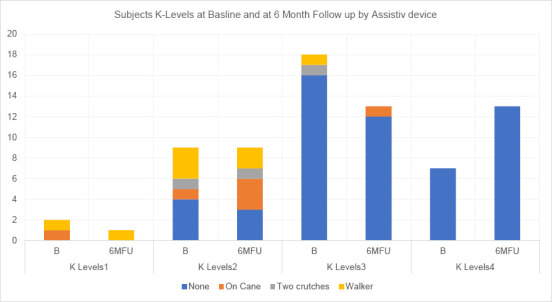
Changes from Baseline to 6MFU in K Levels using the AMPPRO Score, and assistive device use.

The CP noted whether subjects elected to use an assistive device or not when completing the AMPPRO (and TUG) (**Figure 4**). The AMPPRO mobility mean score at baseline was 38 (SD=6.0). Scores increased to 40 (SD=5.4) at 6WFU and remained at 40 (SD=5.5) at 6MFU (**Table 2** and **Figure 4**). Several subjects exhibited improved K level and/or reduced use of assistive devices during the study period:

**Table 2:** Outcome measurements from all subjects (A) of the cohort, with clinical need (B) and without clinical need (C).

A (ALL SUBJECTS)	Baseline^*^ (N=36)	Delivery^**^ (N=36)	6WFU (N=36)	P	6MFU (N=36)	P
**EQ-5D-5L^®^** (Index value)	0.75 (0.18)	-	0.82 (0.15)	<.001	0.84 (0.12)	<.001
Missing		-			2 (5.4%)	
**PLUS-M™** (Percentile)	46.0 (23.6)	-	54.3 (20.6)	<.001	61.4 (16.0)	<.001
Missing		-	2 (5.4%)		1 (2.7%)	
**CLASS** (Percentage)	65.8 (23.1)	84.8 (11.6)	85.1 (13.2)	<.001	87.6 (12.2)	<.001
Missing	1 (2.7%)	1 (2.7%)	2 (5.4%)		1 (2.7%)	
**ABC** (Percentage)	63 (25)	-	75 (15)	<.001	78 (13)	<.001
Missing		-	2 (5.4%)		2 (5.4%)	
**TUG** (sec.)	14.7 (7.4)	14.0 (6.5)	13.0 (5.4)	<.001	12.8 (5.2)	<.001
Missing		2 (5.4%)	4 (10.8%)		1 (2.7%)	
**AMPPRO** (Score)	38.0 (6.0)	-	39.8 (5.4)	<.001	39.6 (5.5)	<.001
Missing	1 (2.7%)	-	3 (8.1%)		2 (5.4%)	
**B (WITH CLINICAL NEED)**	Baseline^*^ (N=26)	Delivery^**^ (N=26)	**6WFU (N=26)**	**P**	**6MFU (N=26)**	**P**
**EQ-5D-5L^®^** (Index value)	0.71 (0.19)	-	0.81 (0.17)	<.001	0.84 (0.14)	<.001
Missing		-			2 (7.4)	
**PLUS-M™(**Percentile)	45.8 (22.2)	-	54.5 (21.3)	<.001	61.2 (15.7)	<.001
Missing		-	2 (7.4)		1 (3.7)	
**CLASS** (Percentage)	62.0 (25.3)	85.4 (11.6)	86.0 (14.7)	<.001	88.2 (12.6)	<.001
Missing	1 (3.7)		2 (7.4)		2 (7.4)	
**ABC** (Percentage)	61 (26)	-	75 (15)	<.001	77 (12)	<.001
Missing		-	2 (7.4)		2 (7.4)	
**TUG** (sec.)	14.2 (7.4)	13.2 (5.5)	13.0 (5.7)	0.08	12.7 (5.3)	0.02
Missing		1 (3.7%)	3 (11.1%)		1 (3.7%)	
**AMPPRO** (Score)	37.8 (6.7)	-	39.6 (5.6)	0.01	39.0 (6.1)	0.02
Missing		-	2 (7.4%)		2 (7.4%)	
**C (WITHOUT CLINICAL NEED)**	Baseline^*^ (N=10)	Delivery^**^ (N=10)	**6WFU (N=10)**	**P Value**	**6MFU (N=10)**	**P**
**EQ-5D-5L^®^** (Index value)	0.83 (0.11)	-	0.84 (0.09)	0.93	0.86 (0.08)	0.50
Missing		-				
**PLUS-M™** (Percentile)	46.5 (28.4)	-	53.8 (20.0)	0.50	62.0 (17.9)	0.06
Missing		-				
**CLASS** (Percentage)	74.2 (14.9)	83.2 (12.3)	83.2 (9.5)	0.03	86.3 (11.6)	<.001
Missing		1 (10.0%)				
**ABC** (Percentage)	68 (22)	-	73 (17)	0.44	81 (15)	0.01
Missing		-				
**TUG** (sec.)	15.9 (7.6)	16.1 (8.8)	13.9 (4.7)	0.02	12.9 (5.1)	0.04
Missing		1 (10.0%)	1 (10.0%)			
**AMPPRO** (Score)	38.8 (5.1)	-	40.3 (5.1)	0.08	41.1 (3.4)	<.001
Missing	1 (10.0%)	-	1 (10.0%)			

All data are presented as mean (SD). Missing data were not included in the statistical tests.

* Baseline measurement regarding existing interface (as a part of the prosthesis).

** Baseline measurement regarding the DS-TF interface (as a part of the prosthesis)

- One K1 subject increased function up to K2 level using 1 cane
- One K2 progressed from a walking frame to 1 cane.
- One K2 who previously used a cane no longer required an assistive device
- One K2 advanced to the K3 level while discontinuing the use of a cane
- One K3 went from currently using a walker to using one cane
- One K3 discontinued the use of two crutches to using no assistive device
- One K2 went from using no aid to using one cane
- 7 subjects (using no aid) increased from K3 to K4
- One subject using no aid declined from K4 to K3 at the 6MFU (**Figure 4**)

**Figure 5:**
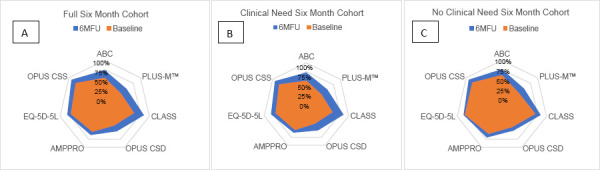
**A:** Illustrating changes (index value from 0-100%) in outcomes for all participants that completed the study from Baseline (Orange) to 6MFU (Blue) for all measures except TUG. **B and C:** Illustrating changes (measured on a scale from 0-100%) in outcomes for the “clinical need” sub-group and the “no clinical need” sub-group (5C), from Baseline (Orange) to 6MFU (Blue) for all measures except TUG.

All outcome measures (incl. OPUS from the previous article) showed significant improvement (**Figure 5A**) for the cohort.

## Satisfaction and functional assessment of participants with the clinical need for new interface (N=26), (Table 2B):

EQ-5D-5L^®^, PLUS-M(tm), ABC, and AMPPRO results in this sub-group are similar to the whole group, showing statistically significant (P=<.001) improvement during the investigation period (**Table 2** and **Figure 5B**). The mean TUG time at baseline on the existing prosthesis was 14.2sec. (SD=7.4). Mean TUG time on the day of fitting with the new interface was 13.2 sec. (SD=5.5) and the time improved further to 13.0 sec. (SD=5.7) at 6WFU and 12.7 sec. (SD=5.3) at 6MFU.

Both TUG and AMPPRO showed statistically significant improvement in function (P=0.02) (**Table 2B**).

## Satisfaction and functional assessment of participants without the clinical need for new interface (N = 10), (Table 2C):

The outcome of EQ-5D-5L^®^ and the PLUS-M(tm) did not reach statistical significance in this sub-group. However, the outcomes directly related to the new interface function (CLASS and AMPPRO) showed significant improvement at 6MFU. Additionally, the mean TUG time in this sub-group at baseline on the existing prosthesis was 15.9sec. (SD=7.6); that is higher than the “clinical need” sub-group. Mean TUG time on the day of fitting with DSTF was 16.1sec. (SD=5.5) and improved to 13.0sec. (SD=5.0) and 12.9sec. (SD=5.1) at 6WFU and 6MFU (P=0.04), respectively, (**Table 2C**).

## DISCUSSION

Aggregate results revealed that the study subjects experienced multifaceted improvements during the study. In the “clinical need” sub-group, the EQ-5D-5L^®^ outcome measure showed significant improvement in the user’s health state compared with the previous interface.

The EQ-5D-5L^®^ mean utility score (0.75), using the existing prosthesis at baseline, increased significantly to 0.84 at 6MFU, indicating that average DS-TF prosthesis users in this study cohort improved their QoL and reached a level similar to the US norm population of 0.85.^37^ The PLUS-M(tm) score at baseline using existing prosthesis was 46, slightly lower than the mean mobility score of 50 reported for the PLUS-M(tm) development sample.^45^ However, the mean mobility scores rose significantly during the study, to 61 at 6MFU (>1 standard deviation above the mean (=10)).

The cohort’s ABC mean score was 63% using the existing prosthesis at baseline, which is in line with findings by Miller-a mean ABC score of 64% among a comparative prosthetic-user population.^39^ However, the score rose significantly above the comparative population to 78% at 6MFU. TUG test times showed significant improvement over the study period, indicating the DS-TF interface did not increase subjects’ risk of falling. OPUS CSS and CSD, also discussed in the previous article,^27^ showed significant improvements. The CLASS outcomes after 6-months showed improvement in all subscales indicating increased user satisfaction with interface stability, suspension, and appearance (**Figure 5A & B**). The AMPPRO mean scores significantly increase from 38 at baseline to 40 at 6MFU. The cohort's mobility improvements were manifested in K-Level increases, reduced dependency on assistive devices and the combination thereof. Notice the cohort's distribution on the graphs in **Figure 4** shift in a positive direction toward increased mobility from baseline to 6MFU. That a socket replacement could have a significant impact on K-level was an unexpected but enlightening outcome.

To our knowledge, few studies have investigated QoL changes using QALY instruments in the prosthetics field. Those that exist were designed to examine the benefits of a knee unit rather than an interface.^54,55^ EQ5D-5L scores in our study reveal a significant increase (p<0.001) in QoL in a relatively large study cohort (for the prosthetics field) attributed to the interface. QoL improvements are the culmination of all the other improvements, including perceived health status, mobility, satisfaction, stability, suspension, comfort, appearance, confidence, balance, pain, and fall-risk. No other known studies have investigated the effects of the TF interface on QoL.

Traditional fitting timelines involve multiple fabrication steps, patient visits, and adjustments.^16^ This study’s certified prosthetists delivered all DS-TF interfaces in a single visit.

This reduces the number of visits typically required to fabricate and deliver a TF socket since DS TF eliminated check sockets for all subjects. There are several reasons why shortening the fitting timeline is important; however, sacrificing quality to gain speed is unacceptable.

There is a glaring absence of published studies on outcomes focusing on fabrication methods and/or different interface designs. One study, by Kahle et al., included 15 subjects comparing 3 socket designs. Results showed no significant difference regarding socket position, movement, or comfort. Kahle et al. stated that skeletal motion within the socket is an important but unquantified factor in outcomes.^56^ No other study with more than 5 subjects was found to compare interfaces regarding design and function over 6 months or demonstrate significant outcome improvements for an interface type.^3^ However, Wurdeman et al. enrolled multiple subjects (N=509), but they did not compare various interfaces. The authors concluded that mobility has a strong positive correlation with both QoL and general satisfaction in lower limb loss patient care.^22^ The absence of comparative studies could be due to a lack of good testing methodology (i.e., lack of sensitivity) and/or a lack of standardized subject inclusion criteria complexity, e.g., patient needs, cause of amputation, surgical technique, skin and soft tissue status/condition, age groups and overall health status.^3^

This study found improvements across multiple outcome measures of both the “clinical need” and “no clinical need” sub-groups. It is noteworthy that users with no perceived need for a socket replacement still experienced significant improvements with DS-TF. As an explanation for this, we might speculate that patients are reluctant to reveal their true thoughts on their current device because they dread the traditionally long fitting procedure and view it as a hurdle that is only worth mounting in certain circumstances. This study shows that DS-TF can sometimes improve patient function and satisfaction regardless of the perceived “clinical need of a new socket”. While this study presents a standardized process for fabrication and delivery of DS-TF, an important question remains: would our field (both practitioners and users) benefit from a more standardized criteria for determining whether or not a patient has a “clinical need” for a new interface? CLASS, as a clinical instrument, could be a viable choice for standardizing socket assessment and replacement. CLASS was developed in 2019 with the intention of being more descriptive than the Socket Comfort Score.^38,57^ Clinicians or investigators may use it to follow prosthesis users’ socket fit and function over time in clinical care or clinical studies, but further research is needed.

Although socket fit is often highlighted in studies as important, the descriptions differ and often lack defining terms.^17^ Thus, there is no indication that “good fit” means the same things to everyone.^14^ There is also a void of investigations on other socket designs measuring the correlation in outcomes regarding mobility and QoL. Of the few studies that look at prosthesis user mobility, most focus on the TT level. If the study does include transfemoral prosthesis users, the focus is on the artificial knee,^22^ or the reference values are still being validated.^58^ Also, as pointed out in the previous article,^27^ interface designs lack standardized descriptions.^17^ Further limitations exist in the disparity of the study subjects. A documented assessment of condition and capabilities following a standardized approach could improve our ability to compare results across different studies.

The present study possesses four central strengths: A comparatively large study cohort (n=47), representative of a relatively normal transfemoral prosthesis user population,^2^ with a comparatively long investigational period (6 months), and using 7 different validated outcome measures. Selecting and combining both subjective and objective measures in this way over a 6-month period gives a broad and deep picture of DS-TF users’ outcomes. We are aware of no other published study on TF interfaces of this size, duration, and scope of outcome measures.

## CONCLUSION

DS-TF users experienced improved QoL, satisfaction, comfort, and function with their new interface, along with increased mobility compared to the previous interface. These findings also revealed that interface replacement with DS-TF could increase objectively measured user K-level, even when socket replacement isn’t clearly clinically indicated. Future studies should include randomized controlled comparisons between different prosthetic interface designs and their impact on prosthetic user QoL and mobility. Additional future studies should contain procedures for identifying a standardized approach to determine when an interface replacement is appropriate.

## DECLARATION OF CONFLICTING INTERESTS

All authors are employees of össur HF except I. F. Atlason. Study principle investigators received no compensation from össur HF.

## AUTHOR CONTRIBUTION

- **Joel Walker:** Conceptualization; Study oversight; Data collection; Writing original; Review and editing.
- **W. Russ Marable:** Conceptualization; Study oversight; Writing original; Review and editing.
- **Christian Smith:** Conceptualization; Data collection; Review and editing.
- **Benedikt Porri Sigurjonsson:** Conceptualization; Obtained funding; Study oversight; Data analysis; Review and editing.
- **Ingi Freyr Atlason:** Data analysis.
- **G. Anton Johannesson:** Conceptualization; Study oversight; Data analysis; Writing original; Review and editing.

## SOURCES OF SUPPORT

This study was financially supported by össur HF.

## ETHICAL APPROVAL

Ethical approval was obtained from Advarra^®^ IRB (CR00128417) and the investigation was registered at Clinicaltrials.gov NCT04312724. Signed informed consent was obtained from all participants.
